# Concordance between culture, Molecular Culture and Illumina 16S rRNA gene amplicon sequencing of bone and ulcer bed biopsies in people with diabetic foot osteomyelitis

**DOI:** 10.1186/s12879-023-08472-w

**Published:** 2023-08-01

**Authors:** Meryl Cinzía Tila Tamara Gramberg, Carmen Knippers, Rimke Sabine Lagrand, Jarne Marijn van Hattem, Marcus Christofoor de Goffau, Andries Edward Budding Budding, Mark Davids, Sebastien Matamoros, Max Nieuwdorp, Vincent de Groot, Martin den Heijer, Louise Willy Elizabeth Sabelis, Edgar Josephus Gerardus Peters

**Affiliations:** 1 Department Internal Medicine, Division of Infectious Diseases, Amsterdam UMC, Vrije Universiteit Amsterdam, De Boelelaan 1117, Amsterdam, The Netherlands; 2 Department of Rehabilitation Medicine, Amsterdam UMC, Vrije Universiteit Amsterdam, De Boelelaan 1117, Amsterdam, The Netherlands; 3 Amsterdam Movement Sciences, Rehabilitation and Development, Amsterdam, The Netherlands; 4 Amsterdam Infection & Immunity, Infectious Diseases, Amsterdam, The Netherlands; 5 Amsterdam UMC Center for Diabetic Foot Complications (ACDC), Amsterdam, The Netherlands; 6 Department of Medical Microbiology and Infection Prevention, Amsterdam UMC, Amsterdam Medisch Centrum, Meibergdreef 9, Amsterdam, The Netherlands; 7 Department of Experimental Vascular Medicine, Amsterdam UMC, Amsterdam Medisch Centrum, Meibergdreef 9, Amsterdam, The Netherlands; 8 Amsterdam UMC, Amsterdam Medisch Centrum, Tytgat Institute for Liver and Intestinal Research, Amsterdam, The Netherlands; 9 Sanger Institute, Cambridge, UK; 10 Inbiome, Amsterdam, The Netherlands; 11 Department of Internal Medicine, Division of Endocrinology, Amsterdam UMC, Vrije Universiteit Amsterdam, Academisch Medisch Centrum, De Boelelaan 1117, Amsterdam, The Netherlands

**Keywords:** Diabetic foot osteomyelitis, Bone and ulcer bed biopsy, Culture, Molecular culture, 16S rRNA gene amplicon sequencing

## Abstract

**Background:**

In clinical practice the diagnosis of diabetic foot osteomyelitis (DFO) relies on cultures of bone or ulcer bed (UB) biopsies, of which bone biopsy is reference standard. The slow growth or fastidious nature of some bacteria, hamper expeditious detection and identification. Rapid molecular techniques may solve both issues, but their additional value for everyday practice is unknown.

We investigated the concordance between conventional culture, the molecular techniques Molecular Culture (MC), and illumina 16S rRNA gene amplicon (16S) sequencing in people with DFO.

**Methods:**

In the BeBoP trial, bone and UB biopsies were obtained from people with DFO who visited Amsterdam UMC. These biopsies were analysed using 1) conventional culture, 2)MC, a rapid broad range PCR analysing the 16S-23S ribosomal-interspace-region, and 3) 16S sequencing, and evaluated concordance among these techniques.

**Results:**

We analysed 20 samples (11 bone and 9 UB) of 18 people. A total of 84 infectious agents were identified, 45 (54%) by all techniques, an additional 22 (26.5%, overall 80.5%) by both MC and 16S, and the remaining 16 species by culture and MC or 16S, or by a single method only. MC and 16S identified anaerobes not detected by culturing in 5 samples, and the presence of bacteria in 7 of 8 culture-negative (6 bone, 2 UB) samples.

**Conclusion:**

The high level of concordance between MC and 16S and the additional ability of molecular techniques to detect various bacteria not detected by culturing opens up prospects for routine use of fast molecular techniques, in clinical settings including DFO.

**Trial registration:**

The BeBoP trial is retrospectively registered on 05–03-2019 in Netherlands Trial Register: NL 7582.

**Supplementary Information:**

The online version contains supplementary material available at 10.1186/s12879-023-08472-w.

## Background

Diabetic foot osteomyelitis (DFO) is a severe infection that is the main cause for lower limb amputation in people with diabetes and foot ulceration, if not expeditiously treated. Rapid clinical identification of all causative bacteria in DFO, needed to make informed choices on targeted antibiotics, is however challenging. The first hurdle is to properly obtain samples, without causing external contamination. Although often used, swab samples are inferior to biopsies for culturing, [1–3] and a positive culture of a percutaneously (or surgically) aseptically obtained bone sample is considered proof of presence of osteomyelitis [4]. Whether culture of bone or ulcer bed biopsies lead to better outcomes is currently under investigation in a large international multicentre BonE BiOPsy (BeBoP) trial [5]. Culturing the obtained samples is currently the reference standard for bacterial detection [4]. Advantages of culturing include the possibility to perform direct microscopy, and to test antimicrobial susceptibility. The antimicrobial susceptibility results allow for targeted antibiotic therapy. Limitations however include that 1) the culturing method relies on bacterial growth, rather than investigating the actual source material, 2) that it takes several days to obtain results, especially with slow growing organisms, and 3) that some (fastidious) bacteria might not grow, and therefore even remain undetected [6–8]. These limitations hamper the speed with which targeted antibiotics can be administered, and might lead to false negative results as bacteria that are present but fail to be cultured go unreported, and thus go untreated. This in turn may result in occult residual infection ultimately increasing the risk of adverse outcomes.

Molecular techniques, especially rapid molecular techniques, could increase the sensitivity of identifying bacterial presence. These techniques do not rely on bacterial growth but detect the presence of bacterial Deoxyribonucleic Acid (DNA) (i.e., source material) directly from a sample. This technique is also advantageous where a sample may potentially contain difficult-to-culture bacteria and may thus contribute to a more rapid identification and treatment of DFO.

One of these molecular techniques is Illumina 16S ribosomal Ribonucleic Acid (rRNA) gene amplicon (16S) sequencing that amplifies a 16S region of the isolated ribosomal DNA by polymerase chain reactions (PCR) targeting hypervariable regions; primers are commonly used to amplify the V1-V2 or V3-V4 regions [9]. This technique has a relatively slow output speed since samples are analysed together (multiplexed) to reduce costs, and molecular analyses of low microbial biomass samples (as is the case with DFO samples) is hampered by reagent and sequencing machine contamination [10].

Molecular Culture (MC) is another molecular technique, and is a relatively new. This rapid technique uses fluorescently labelled PCR primers to differentiate among bacterial species through identification of the length of the 16S-23S rRNA gene Inter Space (IS)-region. With this technique single samples can be analysed yielding results within hours [11]. MC thus has several advantages over 16S sequencing, which importantly include the ability to run single samples instead of batching and to get results within hours and potentially a higher taxonomic resolution. This latter advantage is however dependent on its main disadvantage compared to 16S sequencing which is its dependence on its reference library. 16S representative sequences of ASVs are generated reference library independent and can subsequently be analysed in order to infer their identity. MC requires its own reference library; one which is however expanded continuously. 16S sequencing furthermore normally provides compositional data yet this is of little added value in an infected tissue analysis where the binary analysis for the detection (presence/absence) of infectious agents is the main goal.

In this study we aimed to accurately detect bacteria in (low microbial biomass) DFO bone and ulcer bed (tissue) samples using conventional culturing techniques, and the molecular techniques MC (InBiome bv, Amsterdam, the Netherlands) and 16S sequencing (Microbiota Center Amsterdam, the Netherlands) [9, 11]. Therefore, we compared the results of conventional culturing, MC and 16S sequencing, of bone and UB tissue biopsies from people with DFO participating in the international multicentre randomised controlled BonE BioPsy (BeBop) trial [5].

## Methods

### Bone biopsy (BEBOP) trial

The BeBoP trial is an international multicentre randomised controlled trial, for which inclusions run from 2018 to 2022. In the BeBoP trial we compare the outcomes of DFO treatment based on two diagnostic strategies, percutaneous bone versus ulcer bed biopsy cultures. Only the results of one of these methods was unblinded for the treating physician [5]. At the start of the study both the bone and ulcer bed sample were obtained and cultured. Also, from both samples, material was stored at -80℃. In the present study, we analysed the thawed unblinded samples of the first 18 participants in this BeBoP trial [5].

### Conventional cultures

Bone and ulcer bed samples were examined using conventional culturing techniques for bacteria, according to standard operating procedures of the Amsterdam University Medical Centres laboratory. These procedures included a Gram stain, inoculation of bacteria on Columbia agar + 5% sheep blood (COS) and chocolate agar (PVX) incubated at 35–37 °C under aerobic conditions with carbon-dioxide (CO_2_). On all samples, anaerobic cultures were performed, i.e., inoculation of bacteria on COS incubated anaerobically at 35–37 °C for 4 days. To increase sensitivity, samples were placed in brain–heart infusion broth (BHI), inoculated for 7 days, and examined daily to evaluate growth. In case of bacterial growth in BHI, bacterial subcultures were inoculated on PVX and COS, incubated at 35–37 °C with CO_2_, and under anaerobic conditions.

All morphologically distinct bacterial colonies were characterised to species level with matrix-assisted laser desorption/ionisation time-of-flight mass spectrometry (MALDI-TOF, Bruker Microflex LT, Bruker, London, UK). Antibiotic susceptibility was tested using disk diffusion or Vitek2 (Biomérieux, France), and assessed according to the European Committee on Antimicrobial Susceptibility Testing (EUCAST) clinical breakpoints.

### Molecular diagnostics

We performed molecular diagnostics on the same bone and ulcer bed samples. DNA isolation is the first step for both MC and 16S sequencing.

### DNA isolation

We thawed samples prior to the start of the DNA extraction process. Bone and UB samples were broken down to a piece of 3 × 3 mm and added to a reaction tube with 500 µl Bacterial Shock Buffer 1 (InBiome, IBB23000) and 400 mg Zirconia / Silica beads, 0.1 mm (Biospec, 11079101Z). The samples were vortexed and incubated at 95℃ while shaking at 800 rpm for 10 min. Then 50 µl of Bacterial Shock Buffer 2 (inBiome, IBB24000) was added and tubes were centrifuged shortly. Bead beating was performed for 180 s at room temperature. After bead beating, tubes were centrifuged and supernatant was added to an easyMAG (Biomérieux) container, together with 1 ml of lysis buffer (Biomérieux, Macy l’ Etoile, France) and 1 ml AL buffer (Qiagen, 19,075). We incubated all samples for at least 10 min, before adding 70 µl of Magnetic Silica (Biomérieux). We performed DNA extraction on the NucliSENS easyMAG automated DNA isolation machine (Biomérieux) with the specific protocol, as described by the manufacturer. The DNA was eluted in a 70 µl buffer and stored at 4℃ prior to MC and 16S sequencing.

### Molecular culture 

Molecular culture is a by InBiome patented molecular technique. To perform this technique, we used the isolated DNA to detect and identify bacteria with the CE/IVD marked MC assay (MC ID, inbiome, number: MolCul15000.inBiome). Briefly, we used fluorescently labelled PCR primers to differentiate among bacterial species through identification of the length of the 16S-23S rRNA gene Inter Space (IS)-region. This IS-region was amplified in two multiplex PCRs, using a Veriti 96 PCR system (Applied Biosystems). The first PCR was used to identify species from the Bacteriodetes, Firmicutes, Actinobacteria, Fusobacteria and Verrucomicrobia phyla. The second PCR was used to identify species from the Proteobacteria phylum.

After amplification, we added 20 μl of eMix (inBiome) with 2,5 μl of each of the two PCR products. We used an Applied Biosystems (ABI) Prism 3500 genetic analyser to analyse the fluorescently labelled DNA fragments. We included negative control samples in the analyses to evaluate potential contamination. The genetic analyser yielded fluorescent peaks of specific colour and length that correlate to different bacterial species. The digital data files from the ABI machine were analysed by the automated Molecular Lab Cloud platform (inBiome), which generates bacterial species names and their abundance. When bacteria could not be identified down to the species level because they were not present in the MC reference library, we classified bacteria to the phylum level or in a rare few cases updated the MC refence library when informed by 16S sequencing results [8].

### Illumina 16S r-RNA gene amplicon sequencing

We amplified the 16S region of the isolated ribosomal DNA by PCR targeting the V3-V4 regions using a one-step PCR protocol. We sequenced the 16S amplicons on the Illumina MiSeq platform with V3 chemistry and 2 × 251 cycles. We truncated the forward reads to 240 bases and the reverse reads to 210 bases using USEARCH and inferred amplicon sequence variants (ASV) using UNOISE3. We kept all of these ASVs, in order to visualise patterns of contamination. Furthermore, we ran negative controls from the Molecular Culture analyses to check for contamination. We determined the taxonomy of all ASVs using the RDP classifier and SILVA 16S ribosomal database V132 (genus-level resolution) and additionally manually blasted the main ASVs of interest against the NCBI database for verification and to increase taxonomic resolution where possible.

### Analyses

#### Analyses of conventional culture, Molecular Culture and 16S sequencing results

**Fig. 1:**
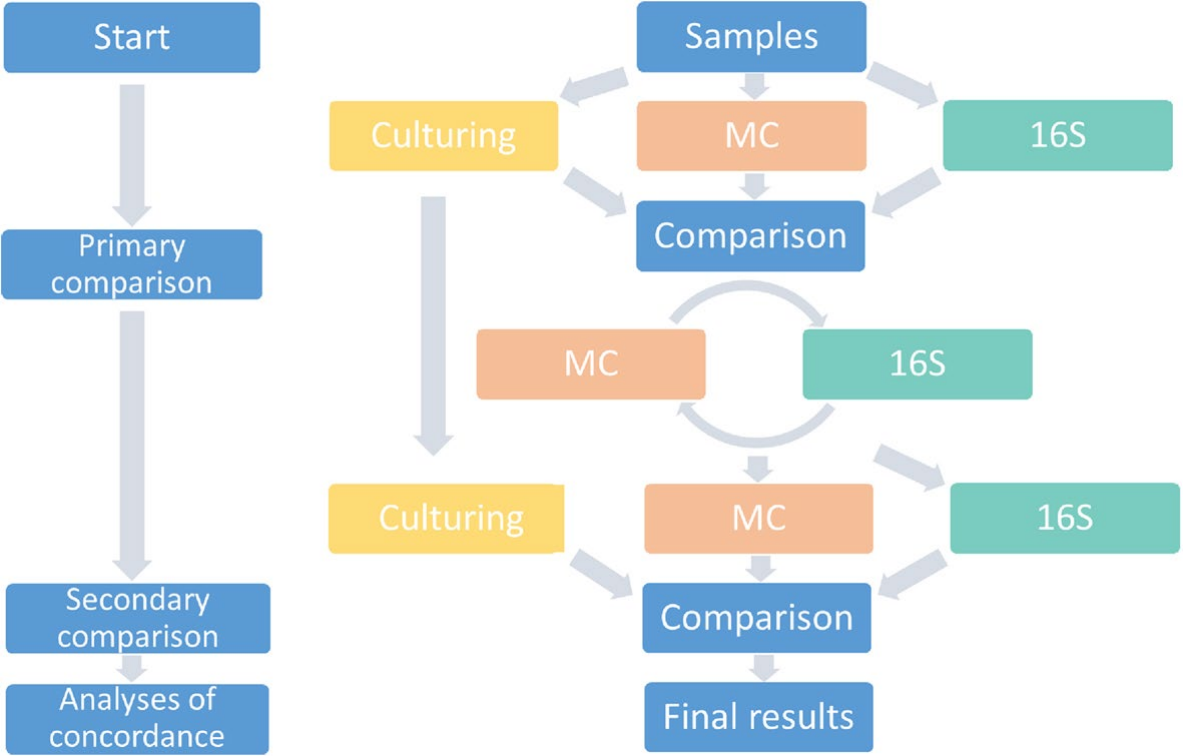
Reciprocal process of analysing results of culturing, Molecular Culture (MC) and Illumina 16S sequencing (16S)

We analysed all samples with conventional culturing, MC and 16S. For the 16S analyses we initially set a 5% threshold of all reads per sample as a lower limit to identify signals of interest. This initial threshold was chosen as bacterial infection in a sample, that should normally be sterile, should typically result in a robust signal. Identification of genuine signals of 16S was furthermore informed and confirmed by culture and MC results whilst MC results were reinterpreted, where needed, by culturing and 16S sequencing results. Figure 1 shows the reciprocal process of comparing results of the different techniques to come to a final identification of bacterial species, in order to evaluate concordance.

### Post hoc analyses and aberrant findings

Before we evaluated concordance we used the results of the primary comparison to perform post hoc analyses on the sequencing data, to retrospectively compare the overall results. We used these post hoc analyses to validate MC signals, to interpret yet undefined MC results, and to refine the MC reference library, to make a better distinction between contaminant and genuine signals with 16S sequencing, and to form an opinion regarding the overall results. We also used the post hoc analyses to interpret and discuss aberrant findings.

### Concordance

We evaluated concordance based on the presence of bacterial identifications per technique per participant. We counted the number of bacterial species and whether they were present in the results of culturing, MC and/ or 16S sequencing, and scored concordance among the techniques.

### Molecular results of culture sterile bone and ulcer bed samples

We examined the results of MC and 16S of the culture sterile bone and ulcer bed samples to get an impression of their additional value for clinical use.

## Results

### Results of culture, MC and 16S sequencing

We analysed 20 samples (11 bone biopsies and 9 ulcer bed) of 18 people. In Table 1 we display the number of bone and ulcer bed samples, and whether these samples provided positive results with one of the diagnostic techniques. Taking the results of all three diagnostic techniques together, we identified 84 infectious agents, represented by 29 different taxonomical groups/species. We grouped bacteria according to their Gram staining and anaerobic characteristics. The number of samples in which each group of bacteria was identified per technique is shown in Table 2.

**Table 1:** Sample results according to diagnostic technique

	Culture	Molecular Culture	Illumina 16S sequencing
Positive results^a^	12	16	16
Positive bone samples^b^	5	9	9
Positive ulcer bed samples^c^	7	7	7

^a^Of 19 bone and ulcer bed samples

^b^Of 11 bone samples

^c^Of 9 ulcer bed samples

**Table 2:** Frequencies of bacterial species found by culture, Molecular Culture and 16S sequencing

Bacterial groups	Bacterial species	Culture	Molecular Culture	Illumina 16S sequencing
Gram positive	*Staphylococcus aureus*	4	4	4
	*Staphylococcus lugdunensis*	2	2	2
	Coagulase-negative staphylococci^a^	1	2	2
	Beta-haemolytic streptococci^b^	2	4	3
	Viridans group streptococci	2	4	3
	*Corynebacterium* spp.	2	3	3
	*Enterococcus* spp.^c^	2	2	2
	*Micrococcaceae*^d^	1	0	1
Gram negative	Enterobacterales^e^	5	5	6
	*Pseudomonas* spp.^f^	2	3	1
Anaerobes		0	5	5
Other^g^		1	3	1

For a complete overview of detected bacterial species we refer to [Suppl. Material]

^a^Except Staphylococcus *lugdunensis*

^b^All were Streptococcus *dysgalactiae*

^c^All were *Enterococcus faecalis* except for one sample where MC could not distinguish between *E. faecalis *and *E. casseliflavus.*

^d^In both culture and 16S sequencing *Kocuria *sp*.* was identified.

^e^Including: *Serratia marscecens*, *Serratia entomophila/nematodiphila*, *Citrobacter* spp., *Klebsiella* spp. and *Proteus* spp.

^f^All were *Pseudomonas aeruginosa* except for one sample where *Pseudomonas *sp. could not be characterised to species level by 16S sequencing.

^g^Mycobacterium sp., Lactobacillus salivarius, Firmicutes spp., Enhydrobacter spp., Haemophilus parainfluenzae

### Post hocanalyses and aberrant findings

Initially, we ignored 16S sequencing signals below the 5% threshold. Their presence was nonetheless sometimes confirmed in the culturing and/or MC results. For example, *Streptococcus* and *Corynebacterium aurimucosum* were detected in one sample by MC and by culturing. These were also detected with 16S, with corresponding signals of 1.6% *Streptococcus* and 3% *Corynebacterium aurimucosum*. Similarly, the presence of *Staphylococcus lugdunensis* (0.5%) and *Streptococcus agalactiae* (1.8%) was confirmed in another sample by 16S. Reciprocally, a few MC analyses were updated, by either investigating smaller peaks in the spectrum in more detail or by adding new strains to its reference library, confirmed by findings found with 16S. Raw data can be found in the supplementary data Sect. 1 and 2 and in the data repository ENA with project number PRJEB56085 (16S). Some large (> 5%) signals of bacteria detected by 16S in could be discarded with a high degree of confidence because they are ecologically implausible and known contaminants [10], and/or because they were not detected using the other 2 techniques. A few bacterial species of some ecological plausibility were only found by MC, e.g., Mycobacterium spp., *Firmicutes* spp., *Lactobacillus salivarius*, and *Haemophilus parainfluenzae*, or by 16S sequencing, e.g., *Enhydrobacter* spp. These are uncommon bacterial species in DFO, not confirmed by either one of the other techniques and were therefore classified as aberrant findings. We could not determine whether these signals were genuine.

### Concordance

**Fig. 2:**
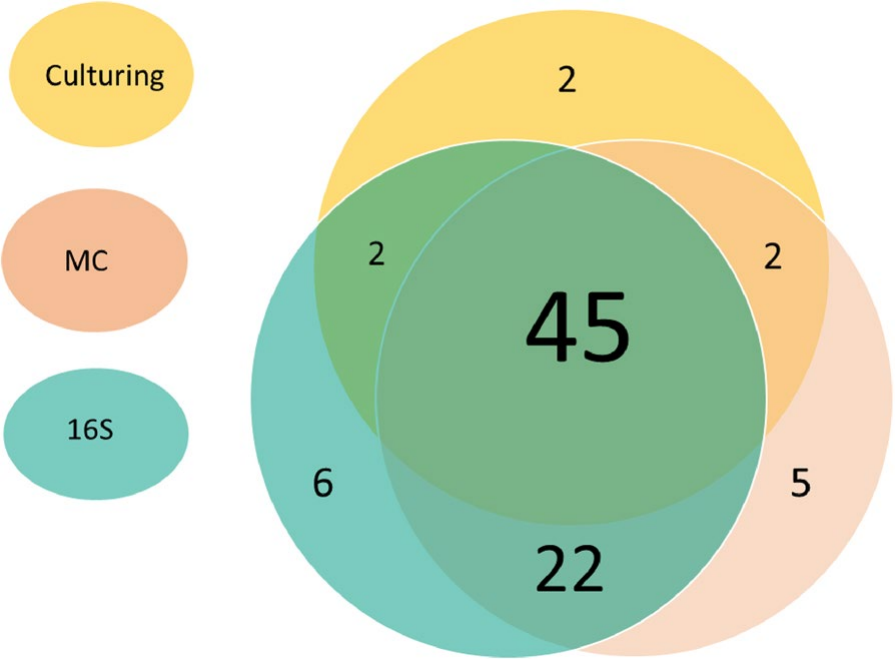
Overview of the concordance between diagnostic strategies in the detection bacterial species: 84 pathogens represented by 29 different bacterial species were detected. *MC* Molecular Culture, 16S Illumina 16S sequencing

Figure 2 shows the number of bacterial species per technique and the concordance among the three diagnostic strategies in a Venn diagram. Of the total of 84 identified species, 13 were identified by a single technique only, the other species were identified by 2 or 3 techniques. There was a high level of concordance between MC and 16S sequencing. The overall concordance between MC and 16S was 80.5%, of which 54% were identified by all techniques and an important additional 26.5% by MC and 16S only (Fig. 2). The most important discordance was with anaerobic bacteria that were both identified by MC and 16S, but not using culturing. Four anaerobic Bacteriodales isolates (*Bacteroides* & *Prevotella*) were only found by MC and 16S (Table 3).

**Table 3:** Anaerobic bacteria found in 4 different samples

Sample type	Anaerobic bacterial species	Molecular Culture	16S sequencing	Concordance
Ulcer bed	*Bacteroides fragilis*	+	-	-
Ulcer bed	*Bacteroides fragilis*	+	+	+
Bone^a^	*Bacteroides fragilis*	+	+	+
Bone	*Prevotella melaninogenica* *Veillonella dispar* *Parvimonas micra*	+ - -	+ + +	+ - -

There were no conventional cultures positive for anaerobic bacteria

^a^Bone cultures of this sample were sterile

### Molecular results of sterile bone and ulcer bed samples

Of the 11 bone samples, 6 had sterile cultures and this was 2 out of 9 for ulcer bed biopsy cultures. Infectious agents were identified in 4 out of the 6 culture sterile bone samples both by MC and 16S (Table 4).

**Table 4:** Results of Molecular Culture and 16S sequencing of 8 sterile cultures

Participant number	Sample type	Culturing	Molecular Culture	Illumina 16S sequencing	Remarks
00005	Bone Ulcer bed	Sterile Sterile	Sterile *Mycobacterium* sp. *Pseudomonas aeruginosa*	Sterile Sterile	MC and 16S had a very low number of reads, identified as contamination Not clear whether the MC signal was genuine MC signal was contamination or signal mix-up
00008	Ulcer bed	Sterile	Sterile	*Enhydrobacter aerosaccus*	Signal of 16S interpreted as contamination
10010	Bone	Sterile	*Serratia* sp*.*	*Serratia* sp*.*	
10011	Bone	Sterile	*Proteus* sp*.*	*Proteus* sp*.*	
00023	Bone	Sterile	*Bacteroides fragilis*	*Bacteroides dorei/fragilis* (post hoc)	Results of 16S are from post hoc analyses
00024	Bone	Sterile	*Staphylococcus* sp	*Staphylococcus pettenkoferi*	

Interestingly, in 2 of the culture negative bone samples that were positive according to MC and 16S, we did identify the same infectious agent (*Serratia marcescens* and *Proteus mirabilis*) when culturing the associated ulcer bed samples of the same participant. It is important to note that we identified additional bacterial species with culturing in the ulcer bed samples. These ulcer bed samples were not analysed by MC or 16S sequencing, since the culture results of these ulcer bed samples belong to unblinded samples in the BeBoP study. All blinded ulcer bed samples will also be allowed to be analysed once the BeBoP trial is finished allowing for the randomisation concealment to be broken.

Of the 9 ulcer bed samples, 2 had sterile cultures (Table 4). One of these samples had positive MC results that could not be reproduced by 16S sequencing, namely a signal matching a *Mycobacterium* spp*.* and a signal matching *Pseudomonas aeruginosa*. Since culturing and 16S sequencing generally detects *Pseudomonas* spp. just fine the latter signal is likely a contaminant or the result of a sample mix-up. Similarly, we could not determine whether the *Mycobacterium* spp*.* signal, as was genuine, since this signal was not confirmed by either one of the other techniques, and the presence of this type of bacteria has not previously been described in DFO in Northwestern Europe.

In the other culture-sterile ulcer bed sample we identified an *Enhydrobacter aerosaccus,* a typical skin bacterium*,* with 16S sequencing. This bacterium is also uncommon in DFO and was likely due to contamination during the 16S library preparation process.

## Discussion

In this study we compared culture results with MC and 16S sequencing results of bone and ulcer bed biopsies of participants with DFO of the BeBoP trial. We found a high level of concordance between MC and 16S sequencing results. Both molecular techniques were able to detect various bacteria that were not detected by culturing in low microbial biomass samples. Importantly, these included many anaerobes. Double verification of bacterial signals not being picked up by traditional culturing methods substantiates the need to further develop fast molecular techniques, such as MC, for routine application in a clinical setting. This rapid molecular technique should, nevertheless, be further validated on larger sample sets. Specifically, the reference library of MC needs to be validated more extensively for use in analysing biopsies of people with DFO. Larger sample sets, including non-infected tissue controls, would also help elucidate whether some of the species associated with the aberrant findings are of genuine bacterial signals. To fully appreciate the added value of a rapid molecular technique such as MC, treatment outcomes of people with DFO should be taken into account as well.

Comparison of clinical outcomes in which treatment is either solely based on culturing results or by MC-derived microbial identification will be the real litmus test. Such a test warrants that antibiotic susceptibitily testing is possible via this molecular technique as well. Broad genotypic antibiotic sensitivity testing is not yet feasible for clinical application, but developments in this field are rapid and it is conceivable that this will also be possible in the near future.

The limitations of 16S sequencing are well known [10]. To reduce costs, 16S samples are typically sequenced multiplexed while it is essential for timely clinical application that samples can be sequenced individually. Also, to identify (reagent) contaminants and genuine signals in 16S data in low biomass samples, it is actually important to sequence multiple samples together (including various negative and positive controls) for comparison purposes. On the other hand, 16S sequencing represents a relatively cheap and excellent complimentary tool for analysing multiple results of other (molecular) techniques e.g., for validation purposes. 16S sequencing has the advantage of having extensive public reference libraries available, but more importantly, has actually become reference library-independent as previously mentioned making it possible to even detect microorganisms that have never been cultured before.

In our study, we found perceived difference between culturing results of bone and ulcer bed biopsies. This is of particular interest because we found that bone samples were more commonly culture negative than ulcer bed samples, and that in the majority of these culture-sterile bone samples MC combined with 16S could convincingly demonstrate the presence of bacterial species. In culture-negative ulcer bed biopsies MC and 16S did not discover any convincing bacterial signals. In addition, signals detected by MC and 16S in certain culture-negative bone samples were detected in some of the corresponding ulcer bed samples. These specific ulcer bed samples, however, also detected additional bacteria not detected by MC or 16S in the corresponding bone sample. This is suggestive of that microbial biomass levels are even lower in bone samples than in ulcer samples, and that bone cultures might not detect all bacteria present, compared with MC and 16S. Also, these findings may imply that ulcer bed biopsies detect various bacteria that are not causative for the actual bone infection. Anaerobic culturing methods, even of ulcer bed samples, do not seem sensitive enough and might require additional testing. The problem of missed bacterial detection in low microbial biomass samples and the problem of missed detection of anaerobic bacteria, can be solved with a DFO validated MC approach. As said before, even with a validated MC approach, the need for culturing currently remains to determine antibiotic resistance profiles and antibiotic sensitivity testing of bacteria. The combination of culturing ulcer bed biopsies and performing MC on bone samples could allow for the most rapid start of guided antibiotic therapy for DFO. MC can quickly identify bacterial species present, whilst resistance profiles acquired via culturing can be used to possibly adjust this therapy.

The BeBoP trial is an ongoing study (follow up phase until the end of 2023), therefore we performed this investigation on a relatively small number of samples. We used this pilot study to explore outcomes, refine our sequencing protocols and improve the MC reference library for bone and ulcer bed biopsies. In a follow up study, we will analyse all samples of the BeBoP study. Samples will then be no longer blinded and we will have access to all culturing information on bone biopsies, matching ulcer bed biopsies and information on participant outcome, possibly allowing us to explain adverse DFO outcomes as the result of previously undetected causative bacteria.

## Conclusion

We found a high level of concordance between the relatively new and fast molecular technique of MC and the established technique of illumina 16S (16S) rRNA sequencing in low microbial biomass samples of bone- and ulcer bed biopsy samples in people with DFO. These molecular techniques were also able to detect various bacteria, including many anaerobes, not detected by conventional culturing in these bone- and ulcer bed samples. The additional value of molecular techniques, opens up prospects for routine use of fast molecular techniques as MC, in diagnosing diabetic foot osteomyelitis and other infections.

## Supplementary Information

**Additional file 1: Supplementary data 1.**

**Additional file 2: Supplementary data 2.**

## Data Availability

All outcome data is reported in Supplementary data nr 1. Additional anonymized data regarding participants of the BeBoP trial can be obtained from the authors (MCTT Gramberg/ EJG Peters) upon reasonable request, and the sequencing data generated and analysed during the current study are available in the ENA repository (weblink: ENA Browser (ebi.ac.uk)), accession number to dataset: PRJEB56085.

## Abbreviations

16S sequencing: Illumina 16S rRNA gene amplicon sequencing
ABI: Applied biosystems
ASV: Amplicon sequence variants
BeBoP trial: BonE BioPsy trial
BHI: Brain-heart infusion
COS: Sheep blood
DNA: Deoxyribonucleic acid
DFO: Diabetic foot osteomyelitis
EUCAST: European Committee on Antimicrobial Susceptibility Testing
IS-region: Inter Space region
MC: Molecular culture
PCR: Multiplex polymerase chain reactions
PVX: Chocolate agar
rRNA: Ribosomal Ribonucleic Acid
UB biopsy: Ulcer bed biopsy
